# Atrophic C2C12 Myotubes Activate Inflammatory Response of Macrophages In Vitro

**DOI:** 10.3390/cells14050317

**Published:** 2025-02-20

**Authors:** Cong Wu, Yishan Tong, Jiapeng Huang, Shuo Wang, Haruki Kobori, Ziwei Zhang, Katsuhiko Suzuki

**Affiliations:** 1 Graduate School of Sport Sciences, Waseda University, Tokorozawa 359-1192, Japan; wucong86@ruri.waseda.jp (C.W.); tongyishan130@ruri.waseda.jp (Y.T.); hjpshidsg1234@toki.waseda.jp (J.H.); wang_sh@akane.waseda.jp (S.W.); koboharu1223@fuji.waseda.jp (H.K.); zhangziwei@fuji.waseda.jp (Z.Z.); 2 Faculty of Sport Sciences, Waseda University, Tokorozawa 359-1192, Japan

**Keywords:** skeletal muscle wasting, macrophage, inflammatory response

## Abstract

Background: Skeletal muscle wasting is commonly observed in aging, immobility, and chronic diseases. In pathological conditions, the impairment of skeletal muscle and immune system often occurs simultaneously. Recent studies have highlighted the initiative role of skeletal muscle in interactions with immune cells. However, the impact of skeletal muscle wasting on macrophage inflammatory responses remains poorly understood. Methods: To investigate the effect of atrophic myotubes on the inflammatory response of macrophages, we established two in vitro models to induce myotube atrophy: one induced by D-galactose and the other by starvation. Conditioned medium (CM) from normal and atrophic myotubes were collected and administered to bone marrow-derived macrophages (BMDMs) from mice. Subsequently, lipopolysaccharide (LPS) stimulation was applied, and the expression of inflammatory cytokines was measured via RT-qPCR. Results: Both D-galactose and starvation treatments reduced myotube diameter and upregulated muscle atrophy-related gene expression. CM from both atrophic myotubes models augmented the gene expression of pro-inflammatory factors in BMDMs following LPS stimulation, including *Il6*, *Il1b*, and *Nfkb1*. Notably, CM from starvation-induced atrophic myotubes also enhanced *Il12b*, *Tnf*, and *Nos2* expression in BMDMs after stimulation, a response not observed in D-galactose-induced atrophic myotubes. Conclusions: These findings suggest that CM from atrophic myotubes enhanced the expression of LPS-induced pro-inflammatory mediators in macrophages.

## 1. Introduction

Skeletal muscle wasting is a widespread condition linked to heightened hospitalizations and disability, thus rendering it a crucial factor in quality of life. It pertains to a decrease in the dimensions of muscle fibers, primarily associated with an imbalance between protein anabolism and protein catabolism in skeletal muscle cells [1]. A wide array of incidents is related to muscle wasting, such as physiological aging, commonly known as sarcopenia, disuse, and chronic diseases [2]. Despite the multifarious origins of skeletal muscle wasting, its pathogenesis appears intimately associated with immunological dysregulation and chronic inflammation [3,4,5,6,7].

Initially, the interaction between inflammatory dysregulation and skeletal muscle wasting was perceived as unidirectional, with research predominantly concentrating on immune cell responses, while overlooking the contribution of skeletal muscle cells [8,9]. However, recent studies have emphasized that skeletal muscle, not only as a crucial locomotor tissue but also as the largest endocrine organ in the human body, actively regulates immunological processes and modulates the inflammatory response [10,11]. Also, sarcopenia appears to be a significant predictor of post-surgery infection. Patients with low skeletal muscle mass exhibit increased vulnerability to infection and a higher risk of mortality due to sepsis, in contrast to individuals with high skeletal muscle mass [12,13]. Though these clinical studies do not demonstrate causality, they indicate a connection between skeletal muscle wasting and a compromised immune response to infections.

The aforementioned studies indicate that skeletal muscle is increasingly acknowledged for its significant involvement in regulating immune processes; nonetheless, the impacts of skeletal muscle wasting on the immunomodulatory capacity, especially the direct regulatory influence on macrophages, remain largely unexplored. Macrophages serve as essential cellular effectors of the innate immune response, initiating an inflammatory response subsequent to tissue damage or infection [14]. Macrophages are pro-inflammatorily activated upon the identification of lipopolysaccharide (LPS) and other pathogen-associated molecular patterns. During this procedure, macrophages release several mediators, including reactive nitrogen species and proinflammatory cytokines, to exert antiproliferative and cytotoxic effects. However, the uncontrolled or dysregulated release of these mediators from hyper-responsive activated macrophages can also lead to tissue damage, potentially facilitating the development of chronic diseases [15]. Given the heterogeneity, plasticity, and tissue-damaging potential of macrophages, it is of great interest to investigate the potential impact of skeletal muscle wasting on the inflammatory response of macrophages.

We established in vitro models of atrophied muscle induced by D-galactose treatment and starvation. D-galactose has been widely used in an in vitro sarcopenia model due to its ability to induce oxidative stress and promote myotube aging [16,17]. Concurrently, serum starvation was utilized as another atrophy model, recognized for its role in diminishing protein synthesis [18]. To investigate the alterations in the immunomodulatory capacity associated with skeletal muscle wasting, we cultured bone marrow-derived macrophages (BMDMs) from mice in vitro with conditioned medium (CM) from atrophied muscle and assessed the expression of inflammation-related genes after stimulation with LPS. This study aims to provide evidence, demonstrating the impact of skeletal muscle wasting on disturbed immune responses.

## 2. Materials and Methods

### 2.1. Culture and Atrophy Induction of C2C12 Cells

C2C12 myoblast cells (RIKEN Cell Bank, Ibaraki, Japan) were cultured at 37 °C under 5% CO_2_ in Dulbecco’s modified Eagle’s medium (DMEM; FUJIFILM Wako, Osaka, Japan) supplemented with 10% fetal bovine serum (FBS; Gibco, Thermo Fisher Scientific, Waltham, MA, USA) and 1% penicillin–streptomycin solution (FUJIFILM Wako, Osaka, Japan). To induce differentiation, the cells were grown to 90% confluence, and then, after being washed with PBS (pH 7.1–7.3; FUJIFILM Wako, Osaka, Japan), the culture medium was replaced with a differentiation medium consisting of DMEM with 2% horse serum (HS; Gibco, Thermo Fisher Scientific, Waltham, MA, USA). The differentiation medium was refreshed every other day. To induce myotube atrophy, C2C12 myoblast cells were plated in 12-well plates at a density of 5.0 × 10^4^/well. After 5 days of differentiation, C2C12 myotubes were divided into 3 groups: a control group (CON, *n* = 4), a D-galactose-treated group (DG, *n* = 4), and a starvation-treated group (ST, *n* = 4). Myotubes in the DG group were treated with 40 mg/mL D-galactose (Sigma-Aldrich, Saint Louis, MO, USA) in differentiation medium for 24 h, as described in previous studies [19,20,21]. Also, for myotubes in the ST group, differentiation medium was replaced with Earle’s balanced salt solution (EBSS; Sigma-Aldrich, Saint Louis, MO, USA) for 7 h, as described in previous studies [22,23], prior to subsequent procedures.

### 2.2. Immunofluorescence Staining and Myotube Diameter Measurement

For immunofluorescence staining, C2C12 myotubes in the CON, DG, and ST groups were washed 3 times with PBS and fixed in 4% paraformaldehyde for 20 min. Then, myotubes were permeabilized in 0.1% Triton-X for 10 min and blocked in 5% BSA for 30 min. The primary and secondary antibodies were as follows: anti-myosin heavy chain (MHC) antibody (recognizes all MHC isoforms) at a concentration of 1:100 (MF20, Development Studies Hybridoma Bank at the University of Iowa, Iowa, USA), and F(ab’)2-Goat anti-Mouse IgG (H + L) Cross-Adsorbed Secondary Antibody Alexa Fluor^®^ 488 conjugate (A11017, Thermo Fisher Scientific, Waltham, MA, USA). Nuclei were counterstained with DAPI (62247, Thermo Fisher Scientific, Waltham, MA, USA). Myotubes were photographed directly in wells under an all-in-one fluorescence microscope (BZ-X800, KEYENCE, Osaka, Japan) using a Plan Apo 10× objective lens and a Plan Fluor 40× Phase Contrast objective lens (Nikon, Tokyo, Japan). The myotube diameter was measured in MHC-stained myotubes, as previously described [24,25,26]. Briefly, in each group, the diameters were measured in a total of 100 myotubes from at least 20 fields that were captured from 4 independent samples. For each myotube, 5 measurements of diameter were obtained perpendicularly to the main axis of the myotube using the ImageJ software (version 1.54f, National Institutes of Health, Bethesda, MD, USA), and the average of these 5 measurements was considered as one single value. Relative myotube diameter was calculated by normalization to the myotube diameter of the CON group.

### 2.3. Collection of Conditioned Medium Derived from Myotubes

After D-galactose and starvation interventions, the medium of myotubes was thoroughly and gently washed 3 times with PBS to remove all the residues, followed by the addition of fresh serum-free DMEM. After 24 h of incubation, the supernatant from each group was collected and centrifuged to remove debris and cells. Subsequently, conditioned medium from the CON group (CM1), DG group (CM2), and ST group myotubes (CM3) was collected after filtering through a 0.22 μm membrane.

### 2.4. Measurement of Lactate Dehydrogenase Release

C2C12 myoblast cells were seeded at a density of 1.5 × 10^3^/well in a 96-well microplate. Cells were then differentiated and introduced to atrophy following the aforementioned procedures. Lactate dehydrogenase (LDH) release was measured using the Cytotoxicity LDH Assay Kit (Dojindo, Kumamoto, Japan), according to the manufacturer’s instructions to assess cytotoxicity.

### 2.5. Harvest and Treatment of Bone Marrow-Derived Macrophages

**Figure 1:**
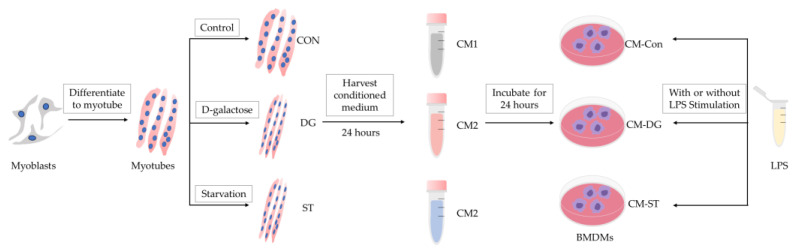
Flow chart including grouping, treatment, and procedure. CON, control myotubes; DG, myotubes treated by D-galactose for 24 h; ST, myotubes starved by incubating in EBSS (Earle’s balanced salt solution) for 7 h. CM1, conditioned medium derived from CON myotubes; CM2, conditioned medium derived from DG myotubes; CM3, conditioned medium derived from ST myotubes; BMDMs, bone marrow-derived macrophages; CM-Con, BMDMs incubated CM1 for 24 h; CM-DG, BMDMs incubated with CM2 for 24 h; CM-ST, BMDMs incubated with CM3 for 24 h; LPS, lipopolysaccharide.

To obtain BMDMs, bone marrow cells were harvested from both sides of the femur and tibia of 9-week-old male C57BL/6 mice (SLC Corporation, Tokyo, Japan) and cultured in a 10 cm cell culture dish at 37 °C under 5% CO_2_ for seven days in DMEM with 10% FBS, 50 ng/mL macrophage colony-stimulating factor (M-CSF; FUJIFILM Wako, Osaka, Japan), and 1% penicillin/streptomycin. The medium was refreshed every other day. Next, BMDMs were plated in a 12-well tissue culture plate at a density of 1.0 × 10^6^/well. Then, BMDMs were divided into 3 groups (CM-Con group, CM-DG group, CM-ST group) and treated with CM1, CM2, and CM3 supplied with 10% FBS, 50 ng/mL M-CSF, and 1% penicillin/streptomycin for 24 h. To evaluate the inflammatory response of BMDMs, 100 ng/mL LPS was added to BMDMs with fresh medium for 1 h after 24 h of conditioned medium treatment (Figure 1).

The present study adhered to the Guiding Principles for the Care and Use of Animals laid out by the Waseda University Institutional Animal Care and Use Committee (approved number: A24-147).

### 2.6. Quantitative RT-PCR Analysis

Total RNA from C2C12 myotubes and BMDMs was isolated using the NucleoSpin RNA Kit (Takara, Kusatsu, Japan), following the protocol given by the manufacturer. The concentration of total RNA was measured by a NanoDrop ND-1000 spectrometer (NanoDrop Technologies, Wilmington, DE, USA). cDNA was synthesized using the PrimeScript™ FAST RT reagent Kit with gDNA Eraser (Takara, Kusatsu, Japan). PCR amplification was conducted with the Thermal Cycler Dice Real Time System (Takara, Kusatsu, Japan) using TB Green^®^ Premix Ex Taq™ II Fast qPCR (Takara, Kusatsu, Japan) under the following conditions: 1 cycle of 95 °C (30 s), 40 cycles of 95 °C (5 s), and 60 °C (10 s). For the analysis of inflammation-related genes, BMDMs were stimulated with 100 ng/mL LPS for 1 h after CM treatment. Relative expression values for target genes were calculated by normalization to the ribosomal protein S18 (*Rps18*) expression. The relative expression levels of the target genes were determined using the 2^–∆∆Ct^ method. The primer sequences for the PCR reactions for each target gene are detailed in Table 1.

### 2.7. Statistical Analysis

Statistical analysis was conducted utilizing IBM SPSS Statistics software (version 29.0.2.0, IBM Corporation, Armonk, NY, USA), and a student’s *t*-test was used to compare the two groups. A two-way ANOVA was applied to examine the effects of LPS stimulation and/or CM from myotubes on the expression levels of inflammation-related genes in BMDMs. Upon identifying a significant interaction, a simple effect analysis was conducted. All data were shown as the mean ± standard deviation (SD), and significance was accepted as *p* < 0.05.

## 3. Results

### 3.1. Characterization of D-Galactose-Treated Myotubes and Starvation-Treated Myotubes

**Figure 2:**
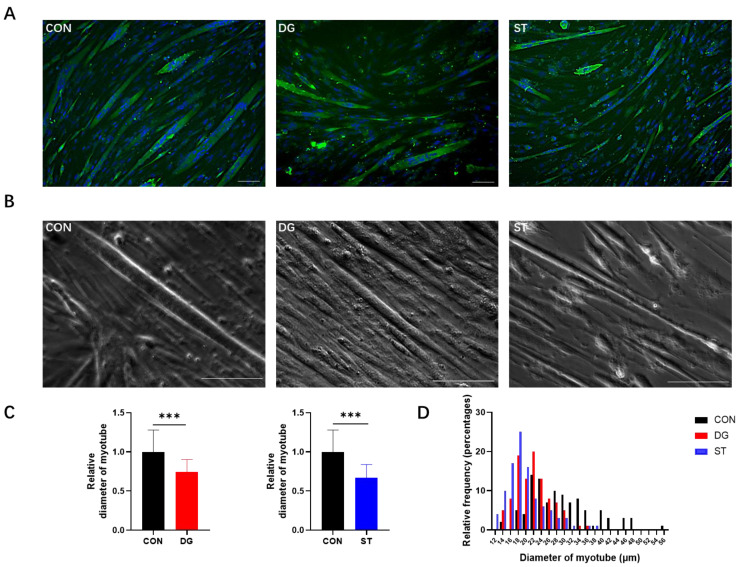
(**A**,**B**) Representative images of (**A**) immunofluorescence staining of MHC (myosin heavy chain) and (**B**) phase contrast microscopy image. Scale bar = 100 μm. (**C**) Relative diameters of myotubes in the DG group (left panel) and ST (right panel) were analyzed based on immunofluorescence staining images. (**D**) Diameter distribution of C2C12 myotubes. Data are presented as means ± SD. *** *p* < 0.001, *n* = 100. CON, control myotubes; DG, myotubes treated by D-galactose for 24 h; ST, myotubes starved by incubating in EBSS (Earle’s balanced salt solution) for 7 h.

In this study, we constructed D-galactose-treated and starvation-treated models to induce myotube atrophy in vitro. We observed myotubes with atrophic changes both in MHC immunofluorescence staining images and phase contrast images (Figure 2A,B). Next, we established these two atrophic myotube models successfully. We measured myotube diameter within MHC immunofluorescence staining images and investigated the expression level of skeletal muscle atrophy-related genes. For the myotube diameter, compared to the CON group, both myotubes in the DG and ST groups showed significantly reduced diameters (Figure 2C,D). The average myotube diameter of CON was 29.29 ± 8.18 μm. After D-galactose and starvation interventions, the average myotube diameter was reduced to 21.80 ± 4.55 μm and 19.64 ± 4.96 μm, respectively. 

**Figure 3:**
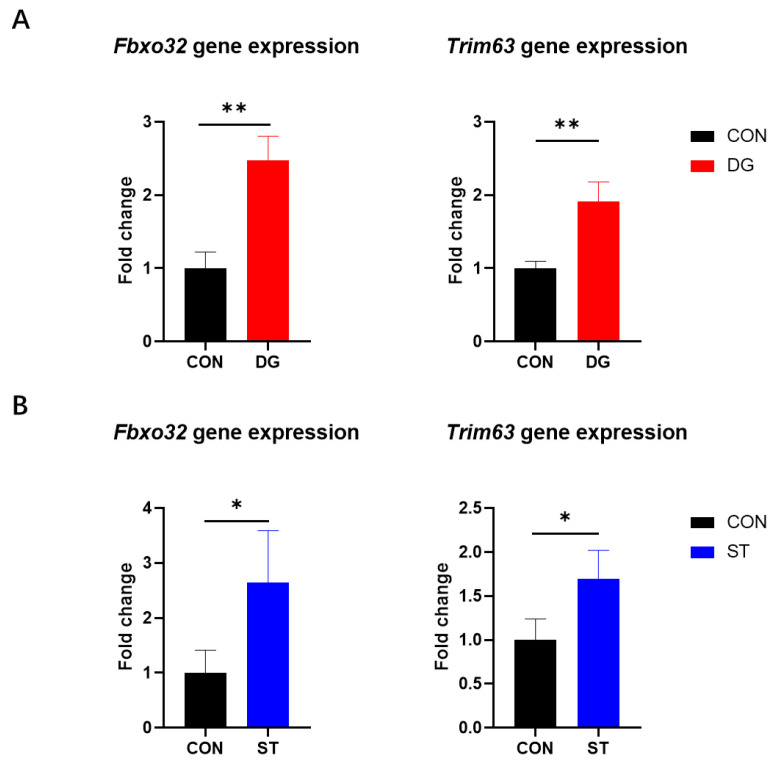
mRNA expression levels of muscle atrophy-related genes, including *Fbxo32* and *Trim63*, in C2C12 myotubes after D-galactose treatment for 24 h (**A**) and starved by incubation in EBSS (Earle’s balanced salt solution) for 7 h (**B**). Data are presented as means ± SD. * *p* < 0.05, ** *p* < 0.01. *n* = 3 for the group in (**A**); *n* = 4 for the group in (**B**). CON, control myotubes; DG, myotubes treated by D-galactose for 24 h; ST, myotubes starved by incubating in EBSS for 7 h; *Fbxo32*, F-box protein 32; *Trim63*, tripartite motif-containing 63.

The ubiquitin–proteasome system is a major process regulating muscle protein breakdown, significantly contributing to muscle atrophy. Muscle RING finger-1 (MuRF-1), encoded by *Trim63*, and muscular atrophy F-box (MAFbx), encoded by *Fbxo32*, are two muscle-specific E3 ubiquitin ligases that have been recognized as crucial regulatory elements in muscle atrophy [27]. Here, we found elevated expression levels of *Fbxo32* and *Trim63* after D-galactose and starvation treatment. Compared to the CON group, the DG group showed 2.47-fold and 1.92-fold higher expression levels of *Fbxo32* and *Trim63*, respectively (Figure 3A; *p* < 0.001). Moreover, the ST group showed 2.65-fold and 1.70-fold higher expression levels of *Fbxo32* and *Trim63*, respectively (Figure 3B; *p* < 0.001). These results demonstrated that both D-galactose treatment and starvation treatment were able to induce myotube atrophy in vitro.

### 3.2. Cytotoxic Effects of D-Galactose Treatment and Starvation Treatment on Myotubes

**Figure 4:**
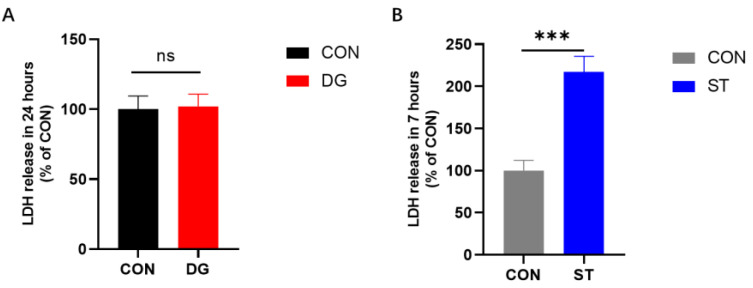
LDH (lactate dehydrogenase) release level after intervention with D-galactose and starvation in myotubes. (**A**) LDH release from untreated myotubes or D-galactose-treated myotubes in 24 h. (**B**) LDH release from untreated myotubes or starvation-treated myotubes in 7 h. Data are presented as means ± SD. ns: no significance observed. *** *p* < 0.001, *n* = 4. CON, control myotubes; DG, myotubes treated by D-galactose for 24 h; ST, myotubes starved by incubating in EBSS (Earle’s balanced salt solution) for 7 h; LDH, lactate dehydrogenase.

LDH is an enzyme that catalyzes the oxidation of lactate to pyruvate, which is rich in myocytes. When cells are damaged, LDH is released into the culture medium due to cell membrane disruption. In this study, we measured LDH levels in the cell culture supernatant to assess cell death after D-galactose and starvation treatments. To account for the different treatment durations, LDH release in the DG group was compared to that in untreated myotubes after 24 h, while LDH release in the ST group was compared to that in untreated myotubes after 7 h. Compared to the CON group, the DG group showed no significant change in LDH release (Figure 4A). However, LDH release was significantly higher in the ST group compared to untreated myotubes (Figure 4B).

### 3.3. Conditioned Medium Derived from Atrophic Myotubes Promotes Lipopolysaccharide-Induced Inflammatory Responses in Bone Marrow-Derived Macrophages

To further explore the effect of atrophic myotubes on macrophage inflammatory responses, we treated BMDMs with CM collected from atrophic myotubes in vitro. Then, after 24 h of treatment, BMDMs were stimulated using 100 ng/mL LPS for 1 h to establish an inflammation model. Next, the expression levels of the inflammation-related genes of BMDMs with or without LPS stimulation were measured, including interleukin 6 (*Il6*), interleukin 1 beta (*Il1b*), interleukin 12b (*Il12b*), tumor necrosis factor (*Tnf*), nuclear factor of kappa light polypeptide gene enhancer in B cells 1 (*Nfkb1*), nitric oxide synthase 2 (*Nos2*), and interleukin 10 (*Il10*).

**Figure 5:**
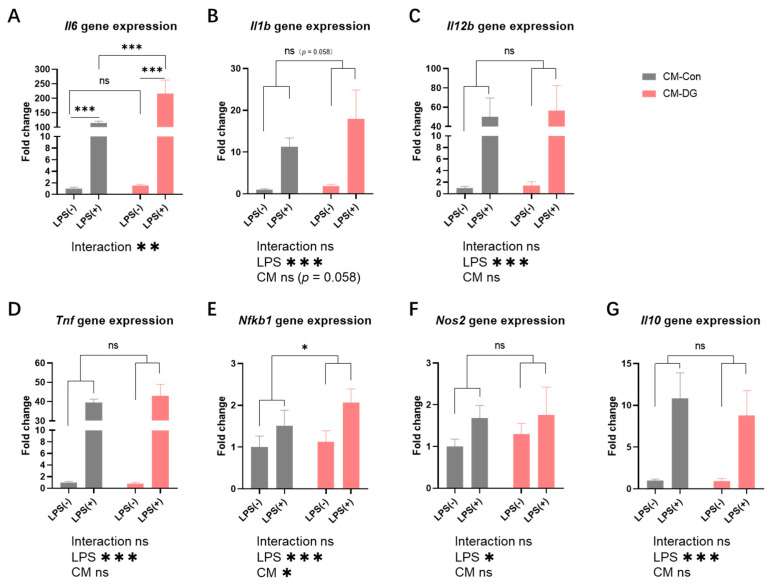
Inflammation-related gene expression in BMDMs (bone marrow-derived macrophages) incubated with control and D-galactose-treated myotube-derived CM (conditioned medium) with and without stimulation (**A**–**G**). Values are the means ± SD. ns: no significance observed. * *p* < 0.05, ** *p* < 0.01, *** *p* < 0.001, *n* = 4. LPS, lipopolysaccharide; CM, conditioned medium; *Il6*, interleukin 6; *Il1b*, interleukin 1 beta; *Il12b*, interleukin 12b; *Tnf*, tumor necrosis factor; *Nfkb1*, nuclear factor of kappa light polypeptide gene enhancer in B cells 1; *Nos2*, nitric oxide synthase 2; *Il10*, interleukin 10. CM-Con group, BMDMs incubated with CM from control myotubes for 24 h; CM-DG group, BMDMs incubated with CM from D-galactose-induced atrophic myotubes for 24 h.

**Figure 6:**
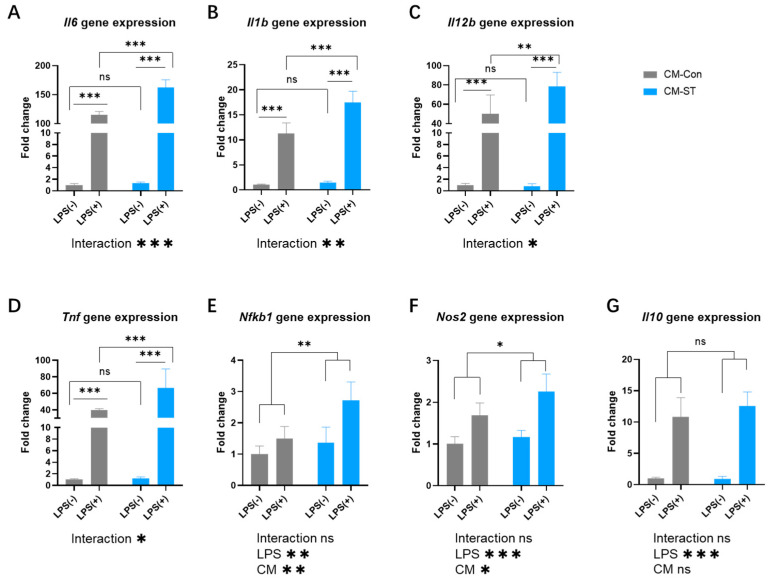
Inflammation-related gene expression in BMDMs (bone marrow-derived macrophages) incubated with control and starvation-treated myotube-derived CM (conditioned medium) with and without stimulation (**A**–**G**). Values are the means ± SD. ns: no significance observed. * *p* < 0.05, ** *p* < 0.01, *** *p* < 0.001, *n* = 4. LPS, lipopolysaccharide; CM, conditioned medium; *Il6*, interleukin 6; *Il1b*, interleukin 1 beta; *Il12b*, interleukin 12b; *Tnf*, tumor necrosis factor; *Nfkb1*, nuclear factor of kappa light polypeptide gene enhancer in B cells 1; *Nos2*, nitric oxide synthase 2; *Il10*, interleukin 10. CM-Con group, BMDMs incubated with CM from control myotubes for 24 h; CM-ST group, BMDMs incubated with CM from starvation-induced atrophic myotubes for 24 h.

For the LPS-treated BMDMs inflammation model, the results are shown in Figure 5 and Figure 6. BMDMs treated with 100 ng/mL LPS showed significantly higher gene expression levels of *Il6*, *Il1b*, *Il12b*, *Tnf*, *Nfkb1*, *Nos2*, and *Il10*, which indicated LPS successfully stimulating inflammatory responses in BMDMs.

After LPS stimulation, compared to the CM-Con group, the expression levels of *Il6* and *Nfkb1* were significantly higher in the CM-DG group (Figure 5A,E). The *Il1b* gene expression level also showed an increasing trend in the CM-DG group (Figure 5B). However, there were no significant changes caused by CM intervention in *Il12b*, *Tnf*, *Nos2*, or *Il10* gene expression (Figure 5C,D,F,G).

Compared to the CM-Con group, the CM-ST group showed significantly higher expression levels of *Il6*, *Il1b*, *Il12b*, *Tnf*, *Nfkb1*, and *Nos2* in stimulated BMDMs (Figure 6A–F). However, there were no significant changes in *Il10* gene expression (Figure 6G). These in vitro investigations revealed that atrophic myotubes elicit a distinct cytokine response in BMDMs upon LPS stimulation.

## 4. Discussion

Skeletal muscle, which makes up 40% of our body mass, has pivotal roles in movement and metabolism [28]. In the past decade, skeletal muscle has been identified as an endocrine organ that releases more than 300 myokines, such as IL-6, IL-7, and IL-15, which affect the immune system [7,29,30]. Serum concentrations of IL-7 and IL-15 diminish with advancing age, correlating strongly with immunological deterioration. Also, in a denervation animal model, mice exhibiting muscular atrophy demonstrated prolonged inflammation and deteriorated sepsis outcomes [11]. In the present study, we used an in vitro model to investigate how skeletal muscle cells affect the immune response of macrophages. Given the multifarious origins of skeletal muscle wasting, we utilized two distinct methods, D-galactose treatment and starvation treatment, to induce myotube atrophy in vitro. Next, we harvested CM from previously mentioned myotubes to co-culture with BMDMs to investigate whether atrophied myotubes are able to impact cell immunomodulation.

Firstly, we incubated fully differentiated myotubes with 40 mg/mL of D-galactose for 24 h or with serum-free EBSS for 7 h to introduce myotube atrophy. D-galactose is an aldohexose found in many foods and is frequently employed as a pharmacological intervention in aging-related research. It is utilized to cause aging both in vivo and in vitro due to its capacity to provoke degenerative alterations in various tissues and organs [31,32,33,34]. Recently, D-galactose has also been used to induce a sarcopenia model because of its capacity to introduce aging-related atrophy in animal skeletal muscle and in C2C12 myotubes [19,35,36]. In line with prior research [17,20], D-galactose treatment increased the gene expressions of Fbxo32 and Trim63, indicating an upregulation of protein degradation in myotubes. Moreover, the immunofluorescence staining of MHC in myotubes also showed decreased myotube diameters in the D-galactose-treated group. At the same time, starvation-treated myotubes presented similar changes in atrophy-related gene expression levels and in myotube diameters, which further support existing data that culturing myotubes with limited nutritional components could lead to rapid protein degradation [22,37,38,39,40,41]. In conclusion, these results indicate that both myotube atrophy models have been successfully established.

Next, the expression levels of inflammation-related genes in BMDMs cultured with CM from control and atrophied myotubes, with or without LPS stimulation, were assessed by RT-qPCR. Some previous investigations have confirmed that C2C12 myotubes may possess the ability to regulate macrophages and their downstream functions [42,43]. The present research is, to our knowledge, the first to investigate the potential impact of atrophied myotubes on the inflammatory response of macrophages in vitro.

Macrophage activation is a plastic process as a consequence of a changing microenvironment. During the defense mechanism against infections, LPS activates a pro-inflammatory response in macrophages via the NF-κB pathway [44,45]. Macrophages first present the M1 phenotype, characterized by pro-inflammatory functions, which involve the secretion of pro-inflammatory cytokines, such as IL-6, TNF-α, and nitric oxide, in response to the stimulation. When the M1 phenotype persists, the overproduction of inflammatory factors produced by macrophages will result in pathology during infections and inflammation [46,47,48]. To determine the effect of atrophy on the immunomodulatory properties of myotubes, we collected CM from atrophied myotubes induced by D-galactose and starvation to co-culture with BMDMs, where untreated myotubes served as the control.

Without LPS induction, the inflammatory response of BMDMs pretreated with a different CM was not significantly different. While LPS stimulation alone elevated the gene expression of *Nfkb1*, it was accompanied by the upregulated M1 macrophage-related downstream gene expression levels of *Il6*, *Il1b*, *Il12b*, *Nos2*, and *Tnf*. These results are generally in line with data reported previously [49,50]. We found it interesting that, in BMDMs, the M1 macrophage-related gene expression levels (*Il6*, *Il1b*, *Il12b*, *Nos2*, and *Tnf*) were significantly elevated by pretreatment with CM from starvation-introduced atrophic myotubes after LPS stimulation. On the other hand, some of them (*Nfkb1* and *Il6*) were elevated by pretreatment with CM from D-galactose-induced atrophic myotubes after LPS stimulation. This is a new finding, indicating that atrophic myotubes may have an immunomodulatory impact on macrophages, exerting the overproduction of pro-inflammatory factors.

Additionally, we investigated the *Il10* expression level in BMDMs. IL-10 is a classical M2 marker that can suppress the pro-inflammatory response by downregulating M1-related cytokines [51,52,53]. However, in the present study, we did not observe that the *Il10* expression level showed the same trend as M1-related gene expression changes caused by atrophic myotubes.

Also, from the above results, we observed that, compared to those cultured with CM from D-galactose-induced atrophic myotubes, BMDMs cultured with starvation-induced atrophic myotubes showed a more radical upregulation in pro-inflammatory genes. Especially in *Il12b*, *Tnf*, and *Nos2*, which are significantly increased after LPS stimulation in BMDMs cultured with starvation-induced atrophic myotubes compared with control myotubes. However, these significant changes did not occur in BMDMs cultured with D-galactose-induced atrophic myotubes. Even though the intervention approach for myotube atrophy is distinct, as a result, myotubes undergoing severer atrophy seem to show a stronger impact on macrophage immune regulation function. However, it is worth mentioning that LDH release analysis indicated that, while a 24 h D-galactose treatment did not induce myotube cell damage, a 7 h treatment significantly increased cell damage. Despite the centrifugation and filtration of CM prior to co-culture to minimize the influence of cell debris, we cannot rule out the possibility that the observed inflammatory response in BMDMs was partially driven by damaged myotubes. From this perspective, D-galactose treatment may serve as a more appropriate approach for inducing myotube atrophy, while minimizing the confounding effects from cell death.

Furthermore, although our gene expression analysis established a clear link between myotube atrophy and macrophage cytokine expression, there are certain limitations in this study. In the future studies, additional protein quantification experiments will be incorporated to further validate the levels of cytokines released by BMDMs. Also, while multinucleated myofibers are the primary components of skeletal muscle, the microenvironment, furthermore, also includes muscle stem cells, immune cells, vasculature, and supportive fibroblasts [54]. Research on skeletal muscle atrophy should not exclusively concentrate on a certain cell type. However, mimicking the atrophied muscle in its entirety seems challenging, underscoring the methodological bias inherent in in vitro research [55]. Nonetheless, in vitro investigations have demonstrated their utility in enhancing the understanding of cell–cell interactions directly and simply. Also, to the best of our knowledge, this is the first study to report that atrophied skeletal myotubes exert a pro-inflammatory effect on LPS-induced macrophages. Moreover, the modulation of pro-inflammatory factors by atrophic myotubes raises the possibility that skeletal muscle wasting may serve as both a clinical predictor and a potential therapeutic target for alleviating disorders associated with macrophage hyperresponsive activation [56]. 

## 5. Conclusions

This study demonstrates that, upon LPS stimulation, atrophied myotubes can enhance the expression of pro-inflammatory mediators, hence modulating macrophage immune responses. These findings suggest that skeletal muscle wasting may contribute to inflammation and could be a therapeutic target for conditions involving macrophage hyperactivation. 

## Figures and Tables

**Table 1:** Primer sequence for real-time PCR analysis.

Gene	Forward (5′–3′)	Reverse (3′–5′)
*Fbxo32*	GCTGGATTGGAAGAAGATG	AGAGAATGTGGCAGTGTT
*Trim63*	CGACATCTTCCAGGCTGCGAAT	ATCACTTCATGGCGGCACGAG
*Il6*	CTTGGGACTGATGCTGGTGACA	GCCTCCGACTTGTGAAGTGGTA
*Il1b*	TGCCACCTTTTGACAGTGATG	ATGTGCTGCTGCGAGATTTG
*Il12b*	GAATGGCGTCTCTGTCTG	GCTGGTGCTGTAGTTCTC
*Tnf*	GTCCCCAAAGGGATGAGAAGT	TTTGCTACGACGTGGGCTAC
*Nfkb1*	GCCTCTAGTGAGAAGAACAA	GTGACCAACTGAACGATAAC
*Nos2*	GCAAACCCAAGGTCTACGTTCA	GAGCACGCTGAGTACCTCATTG
*Il10*	ACATACTGCTAACCGACTCCT	GGCATCACTTCTACCAGGTAA
*R* *ps18*	TTCTGGCCAACGGTCTAGACAAC	CCAGTGGTCTTGGTGTGCTGA

*Fbxo32*, F-box protein 32; *Trim63*, tripartite motif-containing 63; *Il6*, interleukin 6; *Il1b*, interleukin 1 beta; *Il12b*, interleukin 12b; *Tnf*, tumor necrosis factor; *Nfkb1*, nuclear factor of kappa light polypeptide gene enhancer in B cells 1; *Nos2*, nitric oxide synthase 2; *Il10*, interleukin 10; *Rps18*, ribosomal protein S18.

## Data Availability

Data are contained within this article or the Supplementary Materials.

## Supplementary Materials

The following supporting information can be downloaded at: https://www.mdpi.com/article/10.3390/cells14050317/s1, Figure S1: Representative immunofluorescence staining images of MHC and phase contrast images of myotubes.

## Abbreviations

The following abbreviations are used in this manuscript:

BMDMs	Bone marrow-derived macrophages
LPS	Lipopolysaccharide
DMEM	Dulbecco’s modified Eagle’s medium
FBS	Fetal bovine serum
HS	Horse serum
EBSS	Earle’s balanced salt solution
MHC	Myosin heavy chain
CM	Conditional medium
M-CSF	Macrophage colony-stimulating factor
MuRF-1	Muscle RING finger-1
MAFbx	Muscular atrophy F-box
