# Supplementary figures and images for: Atrophic C2C12 Myotubes Activate Inflammatory Response of Macrophages In Vitro
Source: Cells. 2025 Feb 20;14(5):317. doi: 10.3390/cells14050317 (PMC11899532; doi:10.3390/cells14050317)

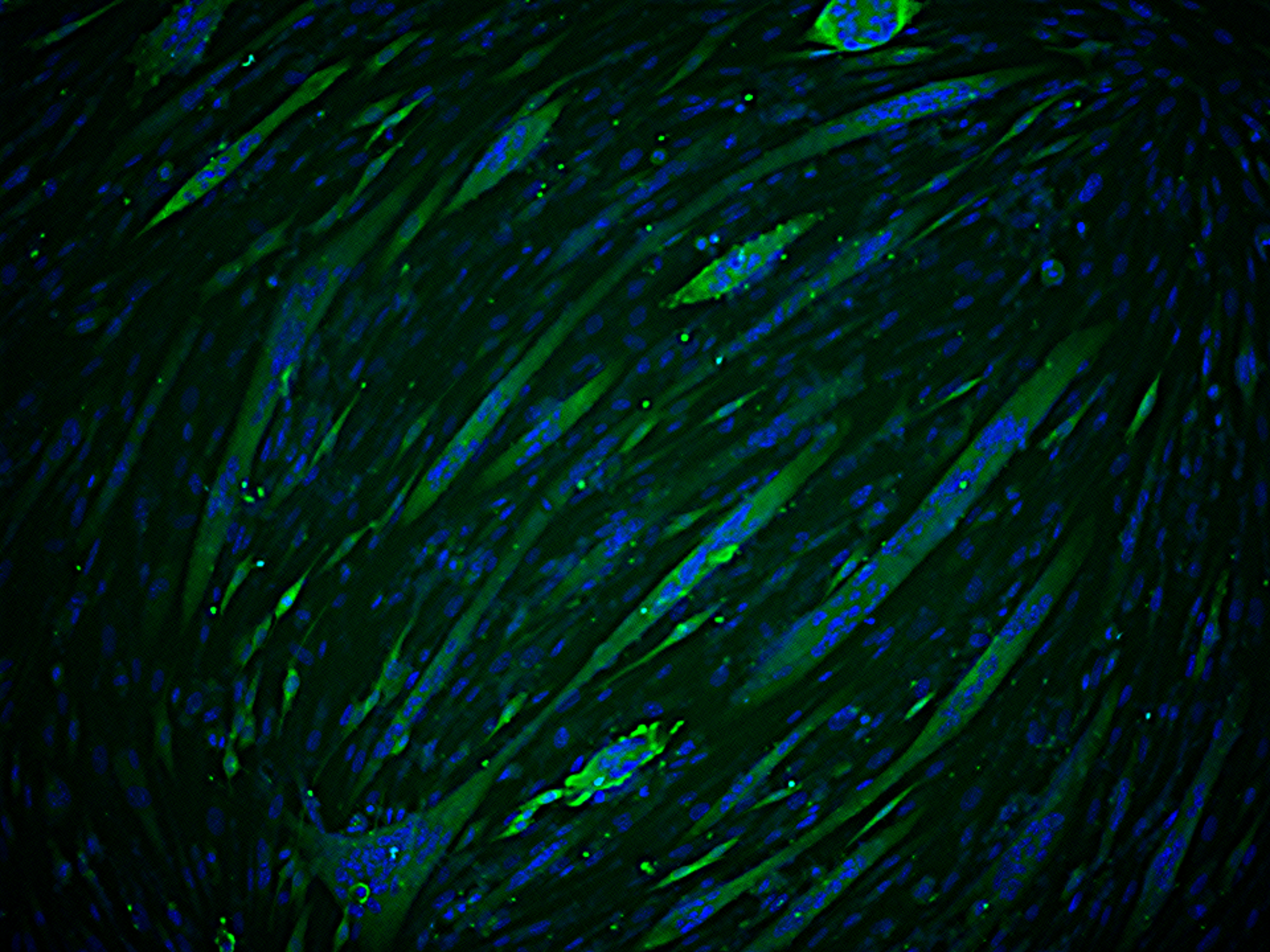

Supplement: Supplementary file 1 [file cells-14-00317-s001.zip › Figure S1. Representative MHC immunofluorescence staining images and representative phase contrast images/CON Representative MHC immunofluorescence staining image.tiff]

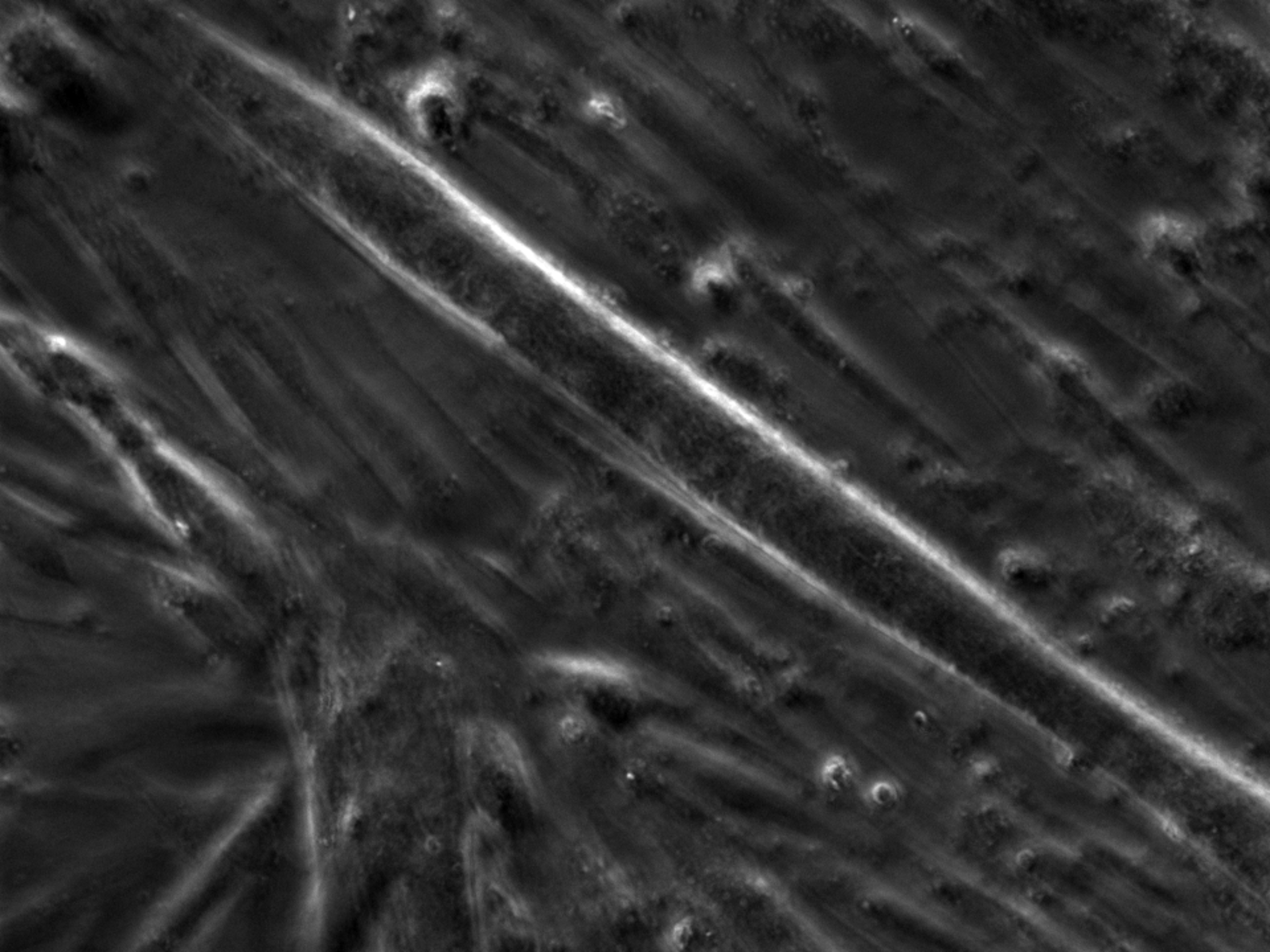

Supplement: Supplementary file 1 [file cells-14-00317-s001.zip › Figure S1. Representative MHC immunofluorescence staining images and representative phase contrast images/CON Representative phase contrast image.tiff]

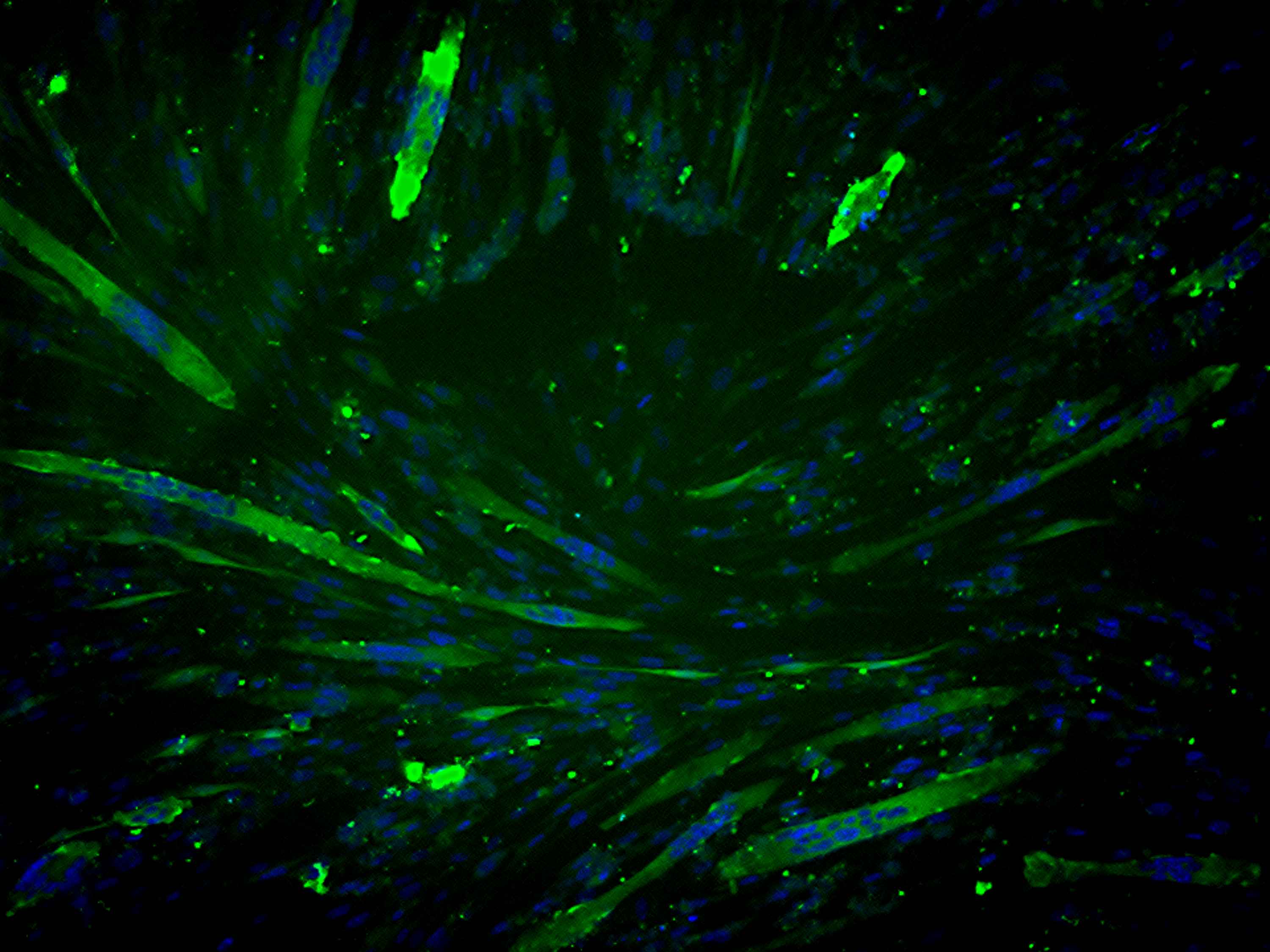

Supplement: Supplementary file 1 [file cells-14-00317-s001.zip › Figure S1. Representative MHC immunofluorescence staining images and representative phase contrast images/DG Representative MHC immunofluorescence staining image.tiff]

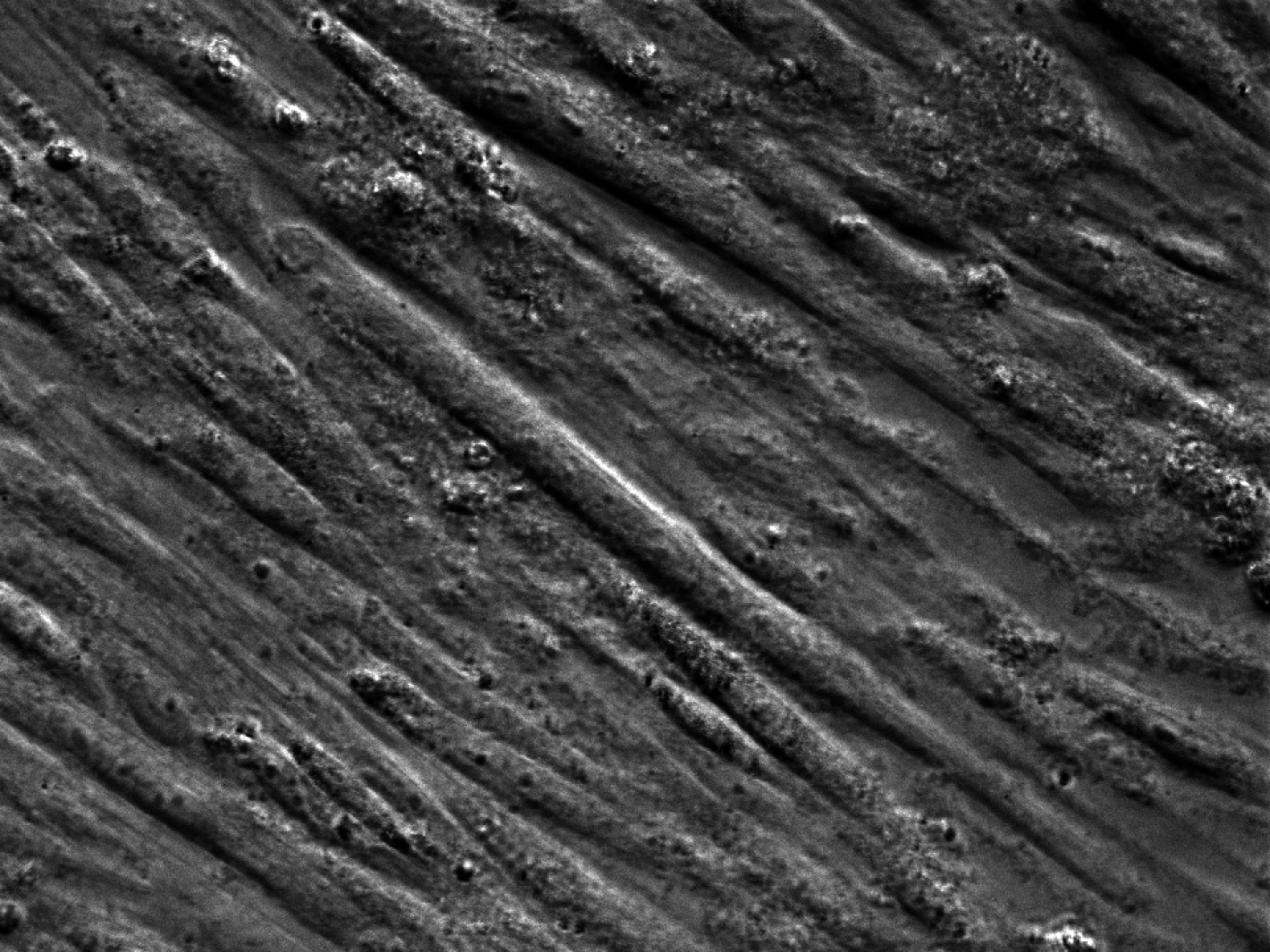

Supplement: Supplementary file 1 [file cells-14-00317-s001.zip › Figure S1. Representative MHC immunofluorescence staining images and representative phase contrast images/DG Representative phase contrast image.tiff]

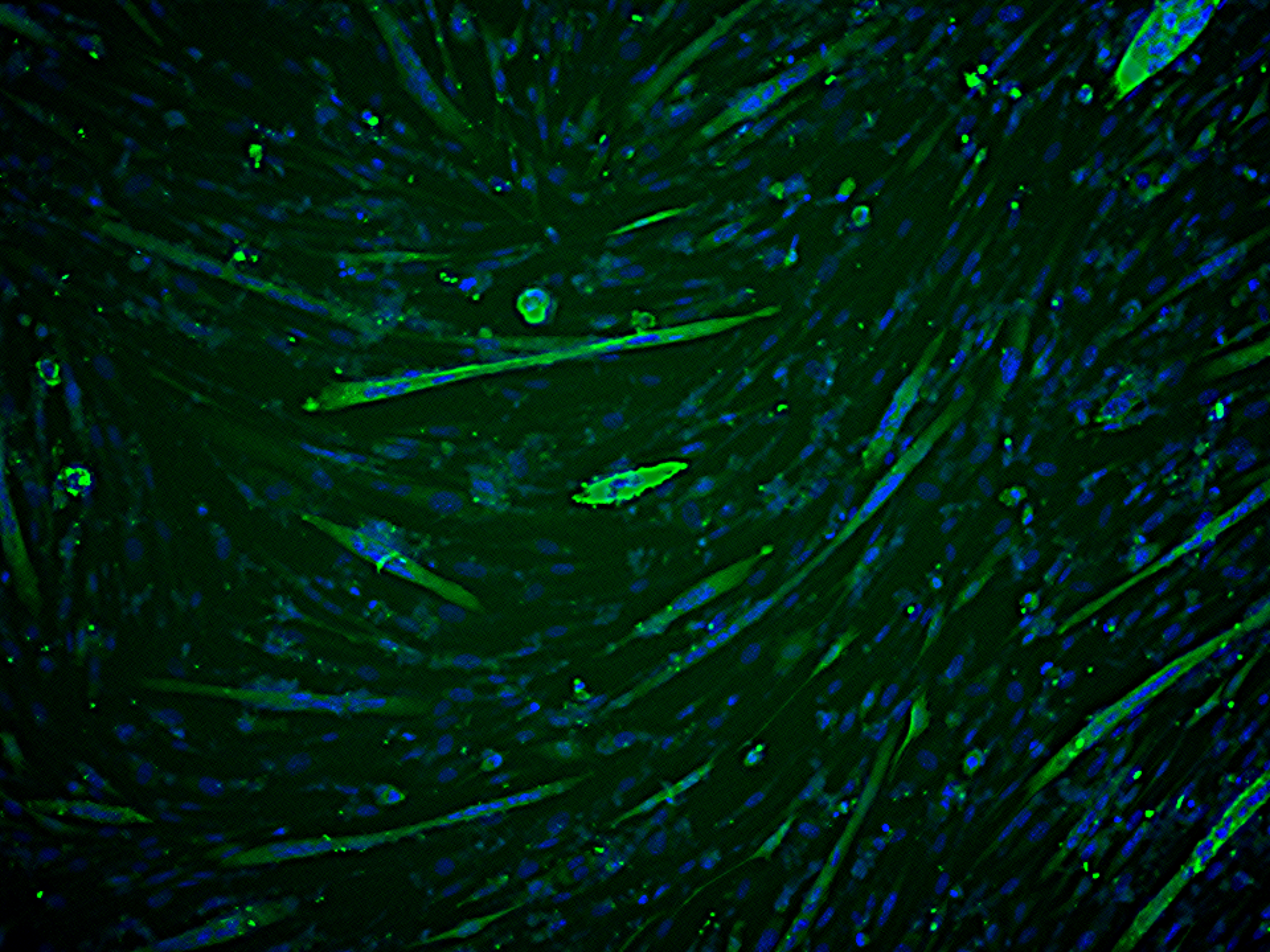

Supplement: Supplementary file 1 [file cells-14-00317-s001.zip › Figure S1. Representative MHC immunofluorescence staining images and representative phase contrast images/ST Representative MHC immunofluorescence staining image.tiff]

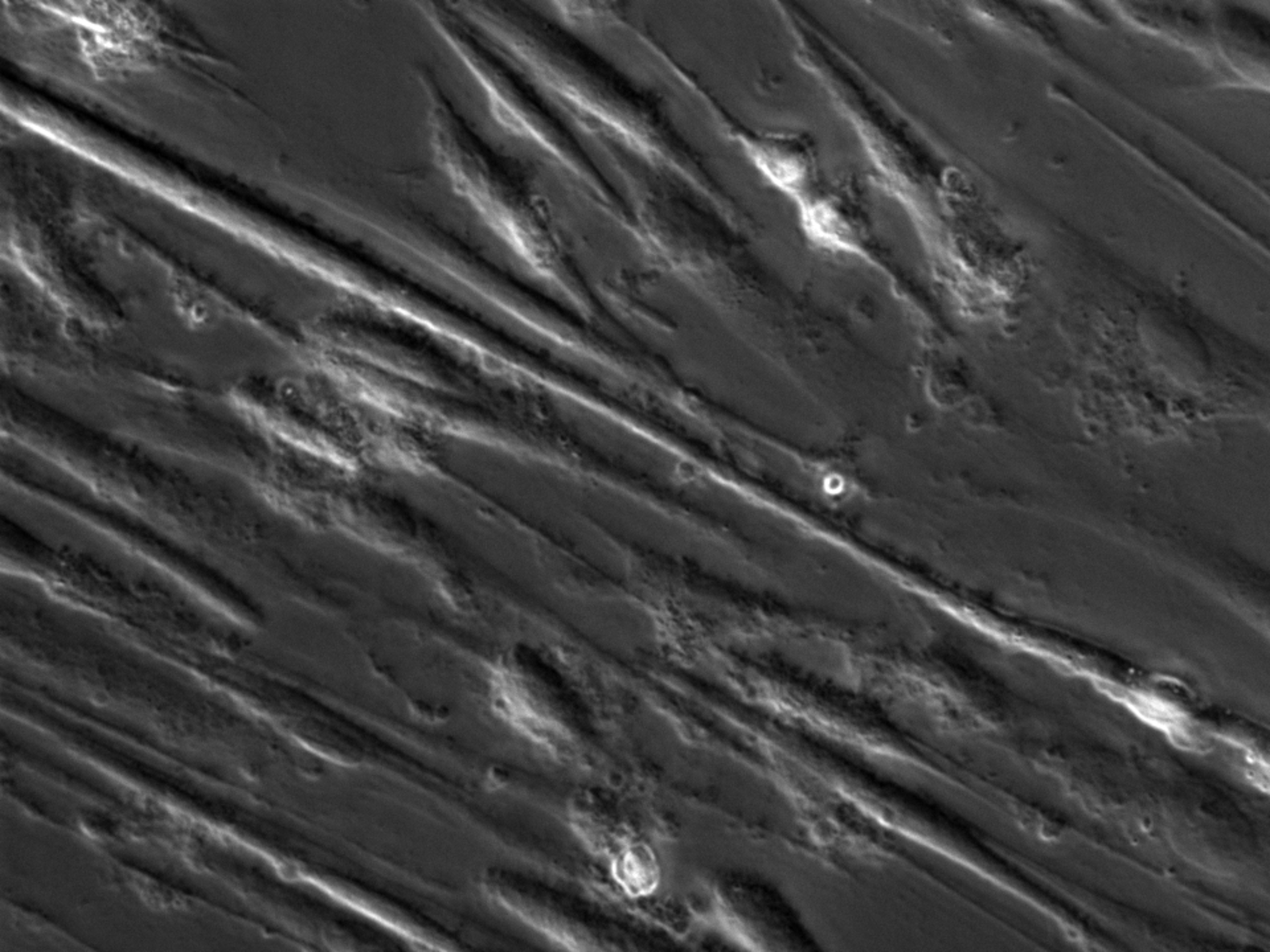

Supplement: Supplementary file 1 [file cells-14-00317-s001.zip › Figure S1. Representative MHC immunofluorescence staining images and representative phase contrast images/ST Representative phase contrast image.tiff]
